# Pararenal aortic pseudoaneurysm secondary to renal stent infection

**DOI:** 10.1016/j.jvscit.2025.101988

**Published:** 2025-09-19

**Authors:** Kelsey R. Lambou, Morgan G. Stadler, B. Seth Decamp, Kara Hessel

**Affiliations:** a Department of Surgery, University of Kansas Medical Center, Kansas City, KS; b Division of Vascular Surgery, Department of Surgery, University of Kansas Medical Center, Kansas City, KS

**Keywords:** Aorta, Pseudoaneurysm, Stent graft, Infection, Nephrectomy

## Abstract

This report presents a case of a 57-year-old woman with protein C deficiency who developed a renal artery covered stent infection owing to *Staphylococcus lugdunensis* infection, resulting in a pararenal aortic pseudoaneurysm. She had previously undergone a thrombectomy and covered stent placement owing to renal artery thrombosis before developing recurrent thrombosis, infection, and a pseudoaneurysm. She required open repair with nephrectomy, aortic debridement, rifampin-soaked Dacron patch angioplasty, and a prolonged antibiotic course. Her anatomy and clinical presentation precluded endovascular options, highlighting the complexity of such cases. This report emphasizes the need for heightened awareness of rare stent infections, individualized surgical planning, and the importance of antibiotic prophylaxis during endovascular procedures.

Seeding and infection is a rare, but serious, complication of endovascular stent placement. *Staphylococcus lugdunensis* is a coagulase-negative staphylococcus (CoNS) first described in 1988 by Freney et al.^1^ Importantly, cultures growing CoNS are often considered contaminants, yet *S lugdunensis* is separated from the group of CoNS by its unique ability to produce severe infection, similar to *S aureus*.^2^ This organism has been known to cause prosthetic joint infections and infective endocarditis. It is most commonly found on the skin in the perineum and inguinal areas, an important factor in the setting of endovascular interventions with groin access.

In this article, we present the case of a 57-year-old woman who developed a renal artery covered stent infection with *S lugdunensis*. Her course was complicated by recurrent stent occlusion and infected pseudoaneurysm development. Ultimately, her infected pararenal aortic pseudoaneurysm was managed with left nephrectomy, excision of all infected aortic wall, and aortic patch angioplasty with rifampin-soaked Dacron. Postoperatively, she was treated with an extended course of intravenous antibiotics and suppressive oral antibiotics. Follow-up imaging demonstrates no evidence of recurrent pseudoaneurysm. Written informed consent was obtained from the patient to share the details of her case before the submission of this case report.

## Case reports

**Fig 1:**
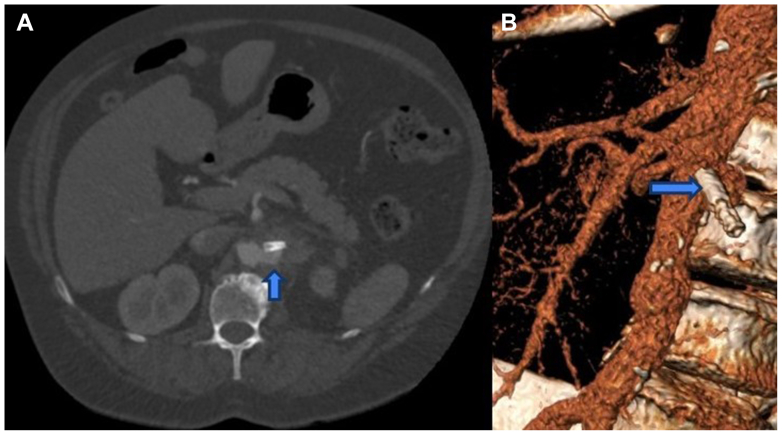
Demonstration of the pseudoaneurysm at the origin of the left renal artery, with indwelling covered stent. **(A)** Axial imaging showing pseudoaneurysm (arrow) posterior to the thrombosed covered stent within the left renal artery. **(B)** Sagittal three-dimensional reconstruction with origin of the left renal artery, thrombosed covered stent and the posterior position of the pseudoaneurysm (*arrow*).

**Fig 2:**
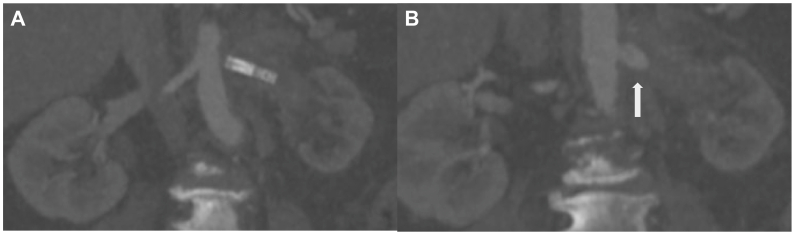
**(A)** Atrophic left kidney with indwelling occluded left renal artery stent. The right renal artery originates at the same level as the left renal artery. **(B)** The pseudoaneurysm (*arrow*) located posterior to the left renal artery and inflammatory changes.

A 57-year-old woman with a notable history of hypercoagulability secondary to protein C deficiency presented as a transfer from an outside hospital owing to worsening left flank pain and fevers in February 2025. Of note, she had known left renal artery thrombosis, requiring two prior endovascular interventions at other hospitals. She first underwent endovascular thrombectomy in February 2024, with subsequent recurrent thrombosis and repeat thrombectomy with angioplasty and covered stent placement in May 2024. She was initiated on apixaban (Eliquis) during the first episode, while ticagrelor (Brilinta) was added after the second occurrence. Further, she had prior hematology work up demonstrating low protein C and antithrombin III activity, with the remainder of the hematology workup unremarkable. In August 2024, she was noted to have complete occlusion of left renal artery on outside imaging and was reported to be taking dabigatran (Pradaxa) alone for anticoagulation. Upon presentation to our hospital, she underwent computed tomography imaging demonstrating an aortic pseudoaneurysm at the left renal artery ostium, with stent erosion into the arterial wall (Fig 1). The left kidney was atrophic and nonenhancing, consistent with known stent occlusion (Fig 2). Owing to her worsening pain and fevers, an infectious component was suspected. Given the small size of her aorta, the location of her right renal artery and superior mesenteric artery (SMA), and the high concern for infection, it was deemed that an open operation would be the most appropriate surgical approach.

The patient underwent a left retroperitoneal surgical approach via a 10th intercostal incision with division of the 10th rib and intercostal muscles, allowing exposure of the supraceliac and distal abdominal aorta. The peritoneum was mobilized medially. The left crus of the diaphragm was divided to fully expose the aorta. After systemic heparinization, proximal and distal aortic control was achieved, including clamping of the SMA and right renal artery. The left kidney was mobilized, the pseudoaneurysm was entered, and the aortic wall was debrided back to healthy tissue. The left kidney, the infected pseudoaneurysm, and the prior covered stent were all removed and sent for microbiology testing. A rifampin-soaked Dacron graft was sewn in place. Perfusion was restored to the SMA, right renal artery, and lower extremities and confirmed by Doppler signals. Hemostasis was achieved with protamine and fresh frozen plasma administration. The diaphragm and abdominal wall were closed in layered fashion. She was taken to the intensive care unit for postoperative care.

**Fig 3:**
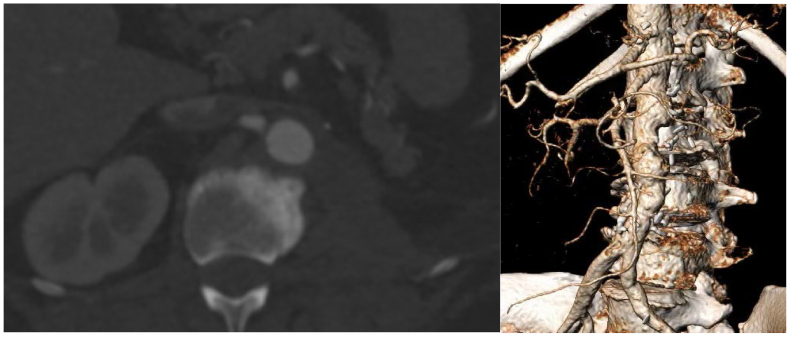
Six-week postoperative computed tomography angiography with three-dimensional reconstruction demonstrates resolution of the pseudoaneurysm following patch repair and left nephrectomy.

Cultures returned positive for *S lugdunensis*, both from the stent and aortic tissue. Her broad-spectrum antibiotics were tapered based on susceptibility testing performed. She was initially treated with a 6-week course of intravenous cefazolin. Upon completion, she transitioned to oral cefadroxil to complete a 6-month course for secondary prophylaxis. A heparin drip was used for anticoagulation in the immediate postoperative period, and she was transitioned back to dabigatran upon discharge, after discussions with hematology. She continues to do well postoperatively and follows up with the hematology and infectious disease teams for ongoing surveillance, in addition to our team. Since the time of surgery, she has undergone repeat imaging demonstrating stability of repair without evidence of pseudoaneurysm recurrence (Fig 3).

## Discussion

Renal artery stent infection with pseudoaneurysm formation is an uncommon complication.^3^ Although rare, there are a few case reports that mirror our findings. Deitch et al^4^ report a case of an infected renal artery pseudoaneurysm with a mycotic aortic aneurysm formation shortly after renal artery angioplasty with stent placement. The patient presented with sepsis and was found to have methicillin-resistant *S aureus* bacteremia. Stent placement was thought to be the seeding source.

*S lugdunensis* is a CoNS bacteria previously regarded as a likely contaminant when found in cultures, much like other CoNS species and normal skin flora.^5^ However, *S lugdunensis* can cause serious and aggressive infections, notably endocarditis, prosthetic joint infections, or, as in the case of our patient, stent graft infections. Our patient had a covered stent placed via access through the left common femoral artery. No perioperative antibiotics were administered, which is standard with percutaneous access. There are varying data on the use of antibiotic prophylaxis in the setting of interventional radiology or endovascular procedures. There is currently no recommendation regarding routine prophylaxis in the setting of stent placement, although consideration should be given in cases of increased risk.^6^ These factors include repeat puncture of the same vessel during a short time interval, use of a sheath that has been in place for more than 24 hours, or prolonged duration of procedure.^7^ This patient had none of these risk factors, so no periprocedural antibiotic was administered at the time of stent placement or thrombectomy at the initial treating facility.

Renal artery pseudoaneurysms can be managed with various approaches, including endovascular or open surgery. Selective arterial embolization or stent graft placement for exclusion or coverage are endovascular options, although open surgical options with primary anastomosis or bypass graft are also available. Decisions regarding salvage of the kidney must also be made. Each of these decisions must be considered in the context of patient-specific anatomy and clinical situations. Our particular case was discussed at a multidisciplinary aortic conference. An endovascular aortic stent graft could be used to fully exclude the left renal artery and pseudoaneurysm, sacrificing the left kidney. The patient's aorta was small, measuring 12 mm. The standard instructions for use of most devices consider 18 to 32 mm an appropriate diameter for the aortic neck landing zone.^8^ Additionally, the right renal artery originated at the same level as the diseased left renal artery and the SMA located only 7 mm superior to this. The instructions for use for most devices requires a neck length of more than 15 mm, which the patient's anatomy did not afford.^8^ Consideration was given to fenestrated or branched endovascular repair, but the aortic diameter limited graft manipulation and cannulation of vessels. These anatomical factors prohibited the ability to successfully deploy an endovascular aortic stent graft while maintaining visceral perfusion. Embolization of the pseudoaneurysm was also considered, but given the involvement of the aortic wall, in combination with the concern for infective process, this technique was deemed inappropriate. Because there was not felt to be an endovascular option that addressed the pseudoaneurysm and maintained visceral perfusion, the decision was made to proceed with an open repair. When deciding on a bypass for a potential treatment option, the need to salvage the kidney was the most important factor. In our specific case, the left kidney had poor flow owing to chronic stent graft occlusion, demonstrated on imaging by atrophy and lack of enhancement with contrast (Fig 2). Our patient's creatinine and glomerular filtration rate were normal, despite the left renal atrophy. Given this, the decision was made not to salvage the left kidney and to proceed with nephrectomy. Therefore, it was determined that the best option would be an open approach with left nephrectomy, exploration of pseudoaneurysm, debridement of infected aortic tissue, and patch repair.

## Conclusions

Infected renal artery pseudoaneurysms are challenging to manage. Endovascular or surgical intervention and antibiotic therapy are the mainstay treatment options. Patient-specific factors such as vessel anatomy may limit repair options and dictate operative planning. Further research is needed regarding the prevention of stent infection and specific guidelines related to perioperative antibiotic use in the setting of endovascular stent placement. Ultimately, early recognition and aggressive management of this uncommon complication are critical because the downstream effects of a stent graft infection can be catastrophic.

## Funding

None.

## Disclosures

None.
